# Differential Effects of Carbohydrates on Behavioral and Neuroelectric Indices of Selective Attention in Preadolescent Children

**DOI:** 10.3389/fnhum.2017.00614

**Published:** 2017-12-20

**Authors:** Anne M. Walk, Lauren B. Raine, Arthur F. Kramer, Neal J. Cohen, Naiman A. Khan, Charles H. Hillman

**Affiliations:** ^1^Department of Kinesiology and Community Health, University of Illinois, Urbana-Champaign, Champaign, IL, United States; ^2^Department of Psychology, Northeastern University, Boston, MA, United States; ^3^Beckman Institute, University of Illinois, Urbana-Champaign, Champaign, IL, United States; ^4^Department of Psychology, University of Illinois, Urbana-Champaign, Champaign, IL, United States; ^5^Center for Learning, Nutrition, & Memory, University of Illinois, Urbana-Champaign, Champaign, IL, United States; ^6^Division of Nutritional Sciences, University of Illinois, Urbana-Champaign, Champaign, IL, United States; ^7^Department of Health Science, Northeastern University, Boston, MA, United States

**Keywords:** carbohydrate, breakfast consumption, event-related potential, P3, glucose facilitation, inhibitory control, attention, cognitive development, NCT02630667 at https://clinicaltrials.gov

## Abstract

The importance of breakfast consumption for ideal cognitive performance has received much attention in recent years, although research on the topic has yielded mixed results. The present study utilized event-related brain potentials (ERPs) elicited during a modified flanker task to investigate the neuroelectric implications of receiving different mixed macronutrient beverages after an overnight fast. A repeated measures design was employed whereby preadolescent participants (9–10 years of age) completed cognitive testing while ERPs were collected during two non-consecutive testing sessions, one in which they received one of three treatment beverages consisting of mixed-macronutrient formulations (either Carbohydrate Blend, Sucrose, Maltodextrin) and the other in which they received a placebo drink containing Sucralose. Performance indices, ERPs, and blood glucose were recorded at three time points before the testing session and after the ingestion of each drink. While the behavioral performance indices and N2 results showed some evidence of glucose facilitation, the effects were small and selective. Participants who received the Maltodextrin treatment showed faster reaction times and more stable N2 amplitudes after ingesting the treatment beverage. The most robust effects were seen in the P3 amplitude measurement. Across the three drink groups, participants showed a marked amplitude increase over time after the placebo drink was ingested, although P3 amplitudes remained stable when a carbohydrate treatment drink was ingested. These effects were eliminated when changes in blood glucose were accounted for, suggesting that the neurolectric effects were directly related to glycemic change. These findings suggest that ingestion of carbohydrates after an overnight fast results in changes to the P3 amplitude of the ERP waveform elicited during an attentional inhibition task.

## Introduction

Children's breakfast habits have received increasing attention in recent years as several reports have posited a relationship between breakfast consumption and academic outcomes (Wesnes et al., 2003; Mahoney et al., 2005; Rampersaud et al., 2005). However, the presumption that the absence of breakfast results in universally negative cognitive consequences lacks consistent support in the literature (Zilberter and Zilberter, 2013). Although ingestion of glucose solutions is thought to facilitate short-term memory and attention, the cognitive implications of consuming a mixed-macronutrient formulation are unclear (Foster et al., 1998; Benton, 2001; Messier, 2004; Hoyland et al., 2008). Further, the neural mechanisms underlying these facilitation effects are underspecified and investigations into these mechanisms have yielded mixed results (de Bruin and Gilsenan, 2009).

Event-related brain potentials (ERPs) are a means of studying the neural correlates underlying cognitive functions by averaging stimulus-locked electroencephalographic (EEG) signals. One of the ERP components, the P3, has been widely studied as a marker of context updating and attentional allocation in a variety of cognitive tasks (Polich, 2007; Riby et al., 2008) in which it is generally assumed that P3 amplitude corresponds to the amount of attentional resources engaged during the cognitive task with larger peaks indicative of greater allocation of attention. P3 latency, on the other hand, is thought to reflect cognitive processing speed, with earlier latencies signifying faster processing (Hoffman et al., 1999). The N2 is a negative-going component that has been studied as a marker of response inhibition, conflict monitoring, and attentional allocation. In these paradigms, a larger (more negative) peak and longer latency occurs with greater stimulus conflict and need for inhibition (Kopp et al., 1996; Heil et al., 2000).

Early studies on the effects of food intake on ERPs showed that meal ingestion had positive effects (i.e., larger) on P3 amplitudes (Geisler and Polich, 1990, 1992a,b, although see Geisler and Polich, 1994). However, subsequent systematic investigations contrasting glucose solutions vs. non-caloric placebo beverages showed mixed effects. For example, a study examining older adults showed a lack of P3 effects (Knott et al., 2001), while studies of young, healthy adults have reported marginal P3 amplitude increases (Hoffman et al., 1999), decreases in P3 amplitude accompanied by earlier onset of P3 latency (Riby et al., 2008), and lack of group differences (Geisler and Polich, 1994) following glucose ingestion. Only when glucose was combined with caffeine have strong P3 facilitation effects been shown (Rao et al., 2005). This effect was accompanied by larger N2 amplitude after participants ingested the treatment drink containing both glucose and caffeine. In short, the extant literature suffers from contradictory results regarding the effect of acute nutritional intake on ERPs, which may be due to methodological differences across studies and the use of different cognitive tasks. Furthermore, the effect of a single bolus of mixed-macronutrient formulation—following an overnight fast—on neuroelectric function remains unexamined in children. This is surprising since children's breakfast has been linked to cognitive and academic performance (Wesnes et al., 2003; Mahoney et al., 2005; Rampersaud et al., 2005).

Furthermore, little is known about how specific nutrient formulations may influence the reported glucose facilitation effect, and although there has been some evidence that carbohydrate formulation may influence selective aspects of cognitive processing, most studies report the use of simple glucose formulations and not complex carbohydrates (Hoyland et al., 2008). Mohd Taib et al. (2012) reported that children performed better on attentional tasks after ingesting an isomaltulose formulation in combination with lactose compared to when they received a standard lactose drink. Additionally, after ingesting the isomaltulose formulation children performed better on a working memory task compared with when they ingested a standard glucose drink. However, Dye et al. (2010) compared ingestion of isomaltulose, sucrose, and water and found that although glycemic profile differed after drink ingestion, measures of working memory and psychomotor performance did not. Similarly, Brindal et al. (2012) altered glycemic load of children's breakfasts by altering the amount of carbohydrate and protein sources and found that while glycemic load differed, cognitive performance did not change across a 3-h window.

Finally, previous work on the neuroelectric implications of glucose facilitation have largely used auditory (Geisler and Polich, 1990, 1992a,b; Hoffman and Polich, 1998) and visual oddball tasks (Geisler and Polich, 1994; Rao et al., 2005; Riby et al., 2008) to examine P3 and N2. While the oddball task has been used extensively to characterize P3 (e.g., Polich and Kok, 1995; Polich, 1996; Goldstein et al., 2002; Conroy and Polich, 2007; Wronka et al., 2008; Horváth et al., 2010; Höller et al., 2013; Verleger and Smigasiewicz, 2016), the P3 has also been widely characterized using the Eriksen flanker task (e.g., Hillman et al., 2004; Clayson and Larson, 2011; Rusnakova et al., 2011; Hsieh et al., 2012). Furthermore, the flanker task has been used to characterize N2 and is considered a task specifically related to attentional inhibition, a marker of cognitive control (Ridderinkhof and van der Molen, 1995; Jonkman et al., 1999; Johnstone et al., 2009; Purmann et al., 2011; Brydges et al., 2014; Groom and Cragg, 2015; Xie et al., 2017).

The primary aim of the present study was to characterize the cognitive and neuroelectric implications of ingesting beverages with varying carbohydrate properties following an overnight fast. To this end, we analyzed the behavioral (response accuracy and reaction time) and neuroelectric (N2 and P3) data of three distinct groups of children, each of whom received a mixed-macronutrient treatment drink while children completed a task of cognitive control. Each group of children served as their own control and received a non-caloric placebo drink containing only sucralose on a separate testing day. A secondary aim was to determine if presumed changes seen in Aim 1 were related to individual responses to changes in postprandial blood glucose, which we measured via area under the curve (AUC). Therefore, we employed the use of three treatment beverages that contained different carbohydrate sources [Carbohydrate Blend (isomaltulose + Fibersol-2®), Sucrose, or Maltodextrin] known to elicit variable glycemic responses due to their different carbohydrate digestion properties (Ohkuma and Wakabayashi, 2001; Foster-Powell et al., 2002). We hypothesized that the cognitive measures would improve following treatment beverages (i.e., faster reaction times and higher performance accuracies) compared to the placebo beverages. Furthermore, we predicted that neuroelectric data would differ following the treatment beverages compared to the placebo beverages, although we left the directionality of these hypotheses open. Finally, we hypothesized that the differences in the treatment and placebo conditions would be explained by the AUC calculation of blood glucose.

## Methods

### Participants

One hundred thirteen preadolescent children recruited from the East-Central Illinois region participated in the study. Participants were recruited from the local community via flyers and email announcements. Participants' guardians completed a phone screening in which they confirmed that their children were between the ages of 9 and 10, were free of diagnosed cognitive and neurological disorders including ADHD, were not taking psychotropic medications, and had normal or corrected to normal vision. Children were excluded if their parent or guardian indicated that they had any milk or soy allergies, were lactose intolerant, or adhered to a strict vegan diet. Additionally, participants were excluded from the present analysis if they were missing behavioral or neuroelectric data on the cognitive tasks (*N* = 20), if they produced ERP data that was not usable (defined by <20 clean ERP trials per condition) (*N* = 3), or if they were considered statistical outliers on any of the dependent measures (*N* = 3). The subsequent sample consisted of 86 children across the three treatment conditions (Carbohydrate Blend: *N* = 27, 10 females; Sucrose: *N* = 26, 14 females; Maltodextrin: *N* = 33, 14 females). All participants gave written assent and their parents or guardians provided written consent before participation in accordance with the University's Institutional Review Board and the Declaration of Helsinki.

### Dietary treatment and placebo

The nutrient breakdown of the treatment and placebo beverages are described in Table 1. The treatment beverages were 8-fluid oz., dairy-based mixed-macronutrient formulations with carbohydrates of varying glycemic properties (Carbohydrate Blend: 68% isomaltulose, 9% maltodextrin, 13% Fibersol-2; Sucrose: 100% sucrose; Maltodextrin: 100% maltodextrin). The placebo drink was a non-caloric beverage artificially sweetened with sucralose. All beverages were served in opaque, unlabeled plastic bottles and were sealed until their consumption.

**Table 1:** Nutrient composition of nutritive treatments and placebo.

**Per 8 oz serving**	**Blend**	**Sucrose**	**Maltodextrin**	**Placebo**
Kcal	150.0	150.0	150.0	0.0
Fats, g	5.0	5.0	5.0	0.0
Proteins, g	7.0	7.0	7.0	0.0
Carbohydrates, g	22.0	22.0	22.0	0.0
Sucrose, g	0.0	17.0	0.0	0.0
Fiber, g	2.5	3.3	3.3	0.0
Maltodextrin, g	1.8	0.0	18.0	0.0
Lactose, g	0.3	0.3	0.3	0.0
Isomaltulose, g	15.0	0.0	0.0	0.0
Sucralose, g	0.1	0.0	0.1	0.1
Water, g	192.6	192.6	192.6	237.0

### Procedure

#### Study design

**Figure 1:**
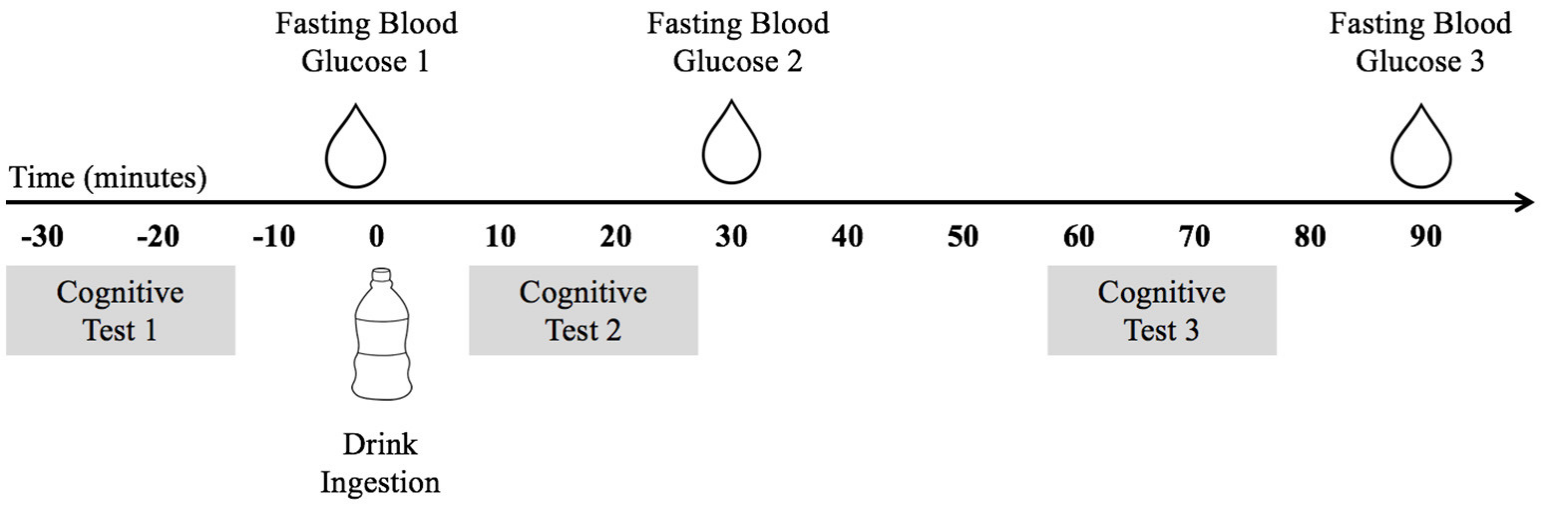
The experimental procedure indicating the timing of blood glucose testing, cognitive testing, and drink ingestion.

These data were collected as part clinical trial NCT02630667. The study utilized a double-blind placebo-controlled trial design. Data were collected over three non-consecutive testing days. On the initial visit, participants underwent a variety of physical measures and completed a demographic and health history survey. On the two subsequent testing days, participants visited the laboratory after an overnight fast of at least 10 h. Figure 1 depicts the experimental procedure. Upon arrival, participants completed the cognitive tasks during the fasted state and then consumed either a mixed-macronutrient formulation or placebo beverage. Follow-up cognitive testing was conducted two subsequent times, ~10 and ~60 min postprandial. A FreeStyle Lite blood glucose monitoring system (Abbott Diabetes Care Inc., Alameda, CA) was used to test participants' blood glucose levels at baseline/fasted, and following the cessation of each cognitive testing session, ~30 and 90 min postprandial. All children were familiarized with the procedure for blood sampling prior to the assent process. Participants were allowed to examine the lancet needles and blood glucose meters and were allowed to decline any testing. The two experimental testing days were counterbalanced and randomized. Both participants and experimenters were blind to the beverage makeup until the completion of the study. All beverages were provided in sealed white bottles with electronic codes as identifiers. While the Sucralose drink used for the Placebo condition tasted sweet and the treatment drinks had milky consistencies, all three of the treatment drinks were very similar in consistency and taste.

#### Cognitive task

A modified version of the Eriksen flanker task (Eriksen, 1995) was used to assess attentional inhibition. In this task, participants viewed a series of visual arrays of fish, each of which had a centrally presented target fish that faced to the right or the left. The target fish was presented amid an array of four task irrelevant fish with two fish flanking each side of the target fish. The participant was instructed to respond to the directionality of the target stimulus with a button press. The task consisted of congruent trials, in which the directionality of the flanking fish was consistent with the target, and incongruent trials, in which the directionality of the flanking fish was opposite that of the target. After receiving instructions, participants completed 50 practice trials (25 of each congruency type). The experimental task consisted of 108 trials, with equiprobable distributions of congruent and incongruent trials as well as left and right target trials, presented in a random order. Stimuli were 2.5 cm illustrations of yellow fish. Each was presented on a blue background for 200 ms. Participants had a response window of 1,550, 1,750, or 1,950 ms, which corresponded to variable inter-stimulus intervals of 1,600, 1,800, or 2,000 ms, respectively.

#### ERP recording

**Figure 2:**
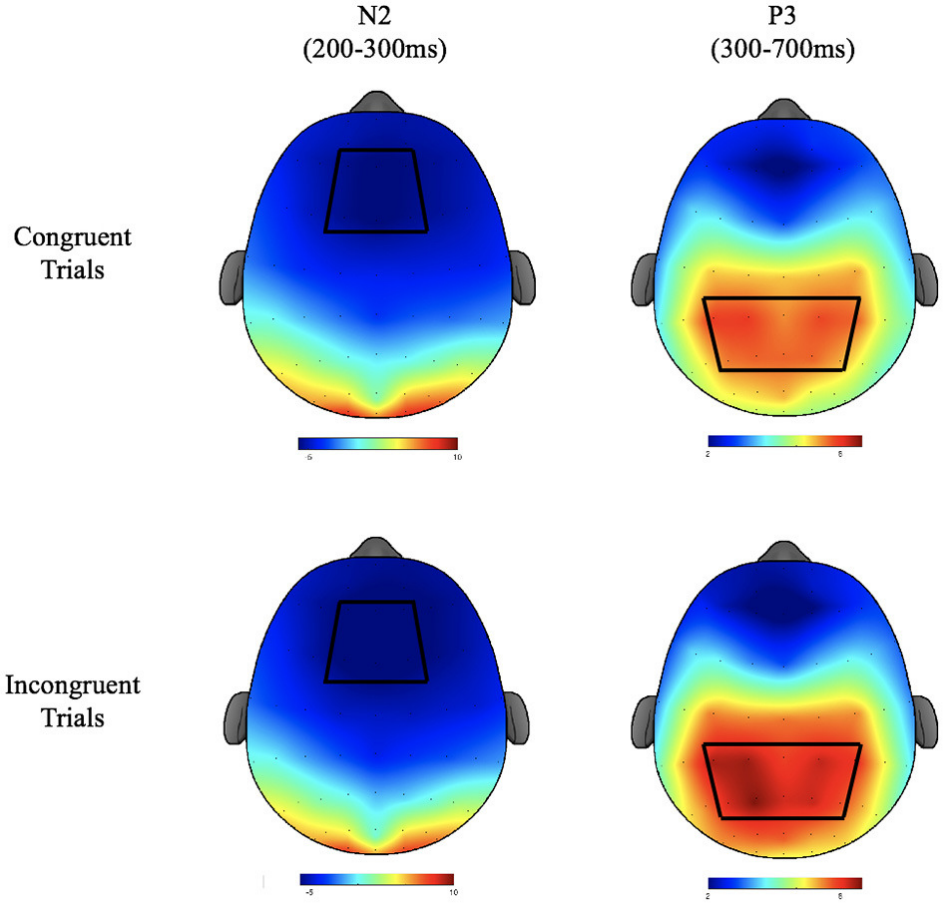
The topographic plots for the N2 and P3 components of the ERP waveform depicted in μV. Bluer regions depict lower amplitudes. Red regions depict higher amplitudes. Regions of interest used for statistical analyses are depicted with heavy black lines.

Electroencephalographic recordings were taken from a 64 channel Neuroscan Quik-cap (Compumedics, Charlotte, NC) with electrode sites arranged according to the international 10–10 system. During recording, electrodes were referenced to an electrode placed between Cz and CPz and the AFz electrode served as the ground electrode. Inter-electrode impedance was kept at <10 KΩ. To monitor electro-oculographic (EOG) activity during recording, additional electrodes were placed above and below the left orbit and outer canthus of each eye. Continuous data were digitized at a sampling rate of 500 Hz, amplified 500 times with a direct current to 70-Hz filter, and a 60-Hz notch filter using a Neuroscan Synamps2 amplifier. Offline, data were re-referenced to the average of the two mastoids (M1, M2). Independent component analysis (ICA) was used to identify eye blink artifacts. If components produced by the ICA met or exceeded a 0.35 correlation with the measured vertical EOG channel, they were considered to be correlated with eye movement and subsequently removed prior to data analysis. Stimulus-locked epochs were created from −200 to 1,200 ms, baseline corrected using the −200 to 0 pre-stimulus interval, and filtered using a zero-phase shift low pass filter at 30-Hz. Trials were excluded from epoched data if they exceeded an artifact detection threshold of ±100 μV or if they were responded to incorrectly (commission and omission errors). The N2 component was defined as the local peak amplitude and corresponding peak latency for the time period occurring from 200 to 300 ms after stimulus onset. Since the morphology of the P3 component showed a less well-defined peak, mean amplitude was calculated for the time window of 300–700 ms after stimulus onset. P3 latency was defined as the time from stimulus onset to the localized peak within the same window. Because scalp topography was not relevant to the primary research aims, ERP data were averaged over a region of interest (ROI) around the topographic maxima of each component (Figure 2). ROIs were based on a grand average of all participants across groups, conditions, and times, following the guidelines put forth by the Society for Psychophysiological Research (Keil et al., 2014). For the N2, the ROI consisted of an average of the F1, FZ, F2, FC1, FCZ, and FC2 electrodes. For the P3 the ROI consisted of an average of CP3, CP1, CPZ, CP2, CP4, P3, P1, PZ, P2, and P4 electrodes.

### Statistical analysis

Statistical analyses were conducted using SPSS version 24 (IBM). A power analysis assuming a medium effect of *f* = 0.25, an alpha level of 0.05, with an objective of achieving power of 0.80, the estimated sample size necessary was 24 participants per drink group. Multivariate outliers were removed by calculating Mahalanobis distance for each condition (placebo, treatment) for each dependent variable. Both behavioral and neuroelectric data were analyzed for each task. Prior to the investigation of the dependent measures of interest, an analysis was performed on blood glucose values. This was done to ensure that pursuing the use of AUC as covariates was justified, as having similar blood glucose values across groups would suggest that inclusion of AUC as covariates would be fruitless. Blood glucose values were submitted to a 3 (Group) × 2 (Condition: Placebo, Treatment) × 2 (Time: 30, 90 min) ANCOVA with the inclusion of blood glucose at baseline as covariates.

Because the study was not a crossover design, the groups were statistically treated independently. Thus, all subsequently described analyses were conducted on each treatment group, respectively. Analyses were conducted on participants' mean reaction times and percent response accuracy to stimuli, and on the amplitude and latency values of the N2 and P3 waveforms of the ERP. Because some groups of participants exhibited different ERP indices at baseline, 2 × 2 × 2 repeated measures ANCOVAs were conducted with repeated measures for Condition (Placebo, Treatment), Time (10 min postprandial, 60 min postprandial) and Congruency (Congruent Trials, Incongruent Trials). Each dependent baseline measure was collapsed over congruency resulting in two baseline metrics (one collected in the placebo condition and one in the treatment condition), which were both entered into the model as covariates. To address our second research aim investigating the effects of blood glucose, AUC was calculated based on the three blood glucose values collected in each condition, yielding one AUC-value for the placebo condition and one for the treatment condition. These values were entered as covariates into a subsequent model along with the baseline measures. Because there were several participants in each group who were unable to give sufficient blood samples, the sample sizes of these groups are slightly reduced (N Carbohydrate Blend = 24; N Sucrose = 22; N Maltodextrin = 29). For all omnibus analyses, F statistics are reported with an alpha level of 0.05 used to determine statistical significance. Partial eta squared values are reported as estimates of effect size. For *post-hoc* analyses, all covariates used in the corresponding omnibus ANOVA were retained and an alpha level of 0.05 was used to determine statistical significance.

## Results

### Demographic data

Participant characteristics are presented in Table 2. Additionally, the means and standard deviations for all performance and ERP indices are presented in Table 3. All non-categorical demographic variables were submitted to Shapiro-Wilk tests of normality within each treatment group. All characteristics were shown to be normally distributed with the exception of BMI, which was shown to be non-normal in all three treatment groups. Across the groups, our sample appeared to be skewed positively with more participants having lower BMI-values. In addition, K-BIT scores were non-normal in the Maltodextrin group (*p* = 0.012). The distribution in this case appears to have a negative skew, suggesting that this group had higher IQ estimates.

**Table 2:** Participant characteristics.

	**Carb blend**	**Sucrose**	**Maltodextrin**
	***N* = 27**	***N* = 26**	***N* = 33**
Age	9.8 (0.6)	10.0 (0.6)	9.9 (0.8)
N reporting	27	26	33
Gender			
(# Females, % sample)	10, 37%	14, 53.8%	14, 42.4%
N reporting	27	26	33
SES			
(# Low, % sample)	4, 14.8%	6, 23.1%	6, 18.2%
N reporting	27	25	32
KBIT (IQ)	113.7 (13.4)	113.8 (11.3)	113.9 (15.2)
N reporting	27	25	30
BMI	17.8 (3.2)	20.6 (6.0)	18.4 (2.9)
N reporting	27	25	30
Blood Glucose for Placebo Condition			
AUC for Placebo	7612 (664)	7288 (563)	7543 (602)
N reporting	24	22	29
Blood Glucose for Treatment Condition			
AUC for treatment	8133 (623)	8184 (904)	8521 (1097)
N reporting	24	22	29

*Means (with standard deviations) reported unless otherwise indicated. Blood glucose AUC reported in mg/dL minutes*.

**Table 3:** Means and Corresponding standard deviations for performance and ERP indices.

	**Placebo**			**Treatment**		
	**Baseline**	**10 min**	**60 min**	**Baseline**	**10 min**	**60 min**
**BLOOD GLUCOSE (mg/dl)**						
Carb Blend	87.1 (5.0)	85.9 (7.2)	81.3 (13.3)	86.0 (4.8)	94.1 (10.6)	87.0 (6.1)
Sucrose	83.8 (6.0)	81.1 (6.2)	79.3 (10.3)	87.0 (6.6)	95.4 (15.2)	86.2 (7.4)
Maltodextrin	84.0 (7.0)	84.8 (8.3)	82.2 (6.6)	83.8 (6.3)	105.5 (19.9)	83.8 (11.2)
**REACTION TIME (ms)**						
**Carb Blend**						
Congruent	520.2 (103.3)	540.8 (103.8)	518.1 (91.8)	513.1 (89.8)	536.5 (104.6)	537.7 (106.3)
Incongruent	565.7 (102.7)	579.8 (110.7)	561.6 (93.8)	557.8 (96.0)	568.4 (98.4)	564.9 (102.1)
**Sucrose**						
Congruent	509.3 (107.2)	524.1 (120.2)	512.3 (111.3)	513.8 (105.4)	518.4 (115.7)	503.5 (104.9)
Incongruent	551.3 (109.2)	554.0 (113.4)	540.8 (110.3)	560.0 (102.8)	549.8 (105.1)	537.1 (110.9)
**Maltodextrin**						
Congruent	547.2 (78.8)	568.1 (92.5)	542.4 (87.6)	538.9 (73.9)	561.9 (91.6)	569.9 (100.2)
Incongruent	587.5 (76.5)	596.0 (86.0)	583.6 (86.8)	586.6 (66.6)	590.7 (84.0)	598.1 (97.9)
**RESPONSE ACCURACY (%)**						
**Carb Blend**						
Congruent	92.9 (5.5)	93.7 (3.8)	92.6 (4.8)	93.2 (5.0)	92.6 (5.3)	90.9 (6.6)
Incongruent	85.2 (8.0)	88.2 (6.1)	87.4 (6.6)	86.6 (8.3)	86.1 (8.4)	85.9 (7.1)
**Sucrose**						
Congruent	94.0 (5.1)	93.2 (6.6)	93.4 (6.9)	94.3 (5.2)	93.1 (5.6)	93.9 (5.0)
Incongruent	87.0 (10.6)	87.3 (8.0)	87.7 (9.0)	88.2 (8.5)	88.2 (6.9)	86.7 (8.7)
**Maltodextrin**						
Congruent	95.5 (3.4)	93.1 (4.7)	93.0 (4.7)	93.1 (6.3)	93.8 (3.7)	91.4 (5.8)
Incongruent	88.1 (6.4)	88.2 (7.8)	88.2 (6.6)	87.8 (9.3)	89.4 (6.0)	86.6 (7.3)
**N2 PEAK LATENCY (ms)**						
**Carb Blend**						
Congruent	264.5 (27.8)	258.1 (26.1)	265.6 (26.9)	264.6 (29.7)	269.2 (29.1)	257.5 (32.0)
Incongruent	261.4 (26.3)	270.1 (22.2)	266.8 (26.6)	262.3 (31.7)	266.1 (29.6)	261.3 (28.4)
**Sucrose**						
Congruent	272.5 (18.0)	271.9 (18.6)	268.7 (24.9)	273.2 (21.4)	270.2 (20.6)	262.2 (24.5)
Incongruent	272.9 (20.6)	273.0 (19.9)	270.7 (21.5)	268.7 (25.6)	269.3 (19.6)	264.8 (24.9)
**Maltodextrin**						
Congruent	261.8 (28.2)	262.2 (29.8)	258.5 (28.9)	267.2 (28.6)	258.8 (31.1)	266.2 (30.0)
Incongruent	259.3 (34.9)	264.4 (28.1)	269.0 (30.7)	268.4 (27.8)	263.2 (30.1)	263.5 (34.3)
**N2 PEAK AMPLITUDE (**μ**v)**						
**Carb Blend**						
Congruent	−12.1 (7.7)	−10.6 (6.1)	−7.6 (5.7)	−9.7 (7.1)	−10.1 (6.9)	−8.1 (6.0)
Incongruent	−12.1 (7.8)	−10.3 (7.6)	−8.7 (6.1)	−10.8 (6.0)	−10.9 (7.3)	−8.8 (6.1)
**Sucrose**						
Congruent	−13.5 (7.5)	−12.8 (6.5)	−10.7 (6.9)	−13.9 (6.4)	−13.5 (7.0)	−11.6 (6.3)
Incongruent	−12.9 (7.5)	−12.7 (6.6)	−11.4 (6.9)	−14.4 (7.3)	−14.2 (6.9)	−12.1 (7.2)
**Maltodextrin**						
Congruent	−11.9 (6.9)	−9.7 (6.3)	−7.2 (6.0)	−10.7 (6.9)	−9.5 (6.5)	−7.9 (7.4)
Incongruent	−11.6 (6.2)	−11.1 (7.1)	−8.1 (5.9)	−11.3 (7.5)	−9.5 (7.1)	−9.6 (6.6)
**P3 PEAK LATENCY (ms)**						
**Carb Blend**						
Congruent	447.4 (99.3)	422.7 (90.1)	417.3 (88.6)	446.9 (94.7)	425.3 (113.4)	414.6 (102.7)
Incongruent	486.4 (91.3)	464.7 (99.3)	436.6 (87.4)	486.6 (90.4)	491.5 (94.8)	461.6 (93.7)
**Sucrose**						
Congruent	458.0 (76.4)	429.4 (103.9)	420.8 (83.7)	423.5 (77.1)	429.9 (100.9)	430.9 (102.5)
Incongruent	473.1 (96.2)	476.0 (102.3)	443.3 (87.2)	472.3 (89.5)	450.7 (107.2)	454.6 (84.4)
**Maltodextrin**						
Congruent	436.1 (90.9)	435.9 (115.6)	398.3 (86.9)	466.3 (111.0)	428.7 (101.2)	453.1 (115.3)
Incongruent	477.9 (83.7)	483.6 (112.7)	460.3 (112.9)	505.7 (87.8)	479.5 (105.5)	442.6 (103.8)
**P3 MEAN AMPLITUDE (**μ**v)**						
**Carb Blend**						
Congruent	9.1 (5.3)	7.4 (4.1)	14.5 (6.1)	8.4 (5.0)	7.2 (5.4)	6.0 (5.2)
Incongruent	8.7 (5.6)	8.2 (4.5)	14.8 (4.9)	9.7 (5.3)	7.7 (5.9)	7.1 (5.9)
**Sucrose**						
Congruent	8.9 (4.7)	6.7 (4.6)	14.1 (5.2)	8.8 (6.0)	5.9 (5.7)	5.9 (5.2)
Incongruent	10.3 (5.3)	8.2 (5.0)	14.8 (5.9)	9.2 (6.7)	6.2 (6.2)	7.2 (5.0)
**Maltodextrin**						
Congruent	8.4 (3.8)	6.9 (3.7)	16.5 (6.0)	10.5 (5.0)	8.0 (5.0)	8.3 (5.3)
Incongruent	9.6 (4.5)	7.2 (3.5)	16.5 (4.8)	11.4 (4.8)	9.2 (4.8)	7.7 (5.8)

Demographic variables were submitted to 1-way ANOVAs. All measures were shown to be similar across groups with the exception of BMI, although this comparison was not significant when accounting for family-wise error [*F*_(2, 82)_ = 3.38, *p* = 0.039]. *Post-hoc* Bonferroni corrected tests confirmed children in the Sucrose group had higher BMIs than children in the Carbohydrate Blend group (*p* = 0.044), and that children in the Maltodextrin group did not significantly differ from the other two groups. Children did not differ in any other characteristics including age, sex, IQ, or socio-economic status.

### Blood glucose data

**Figure 3:**
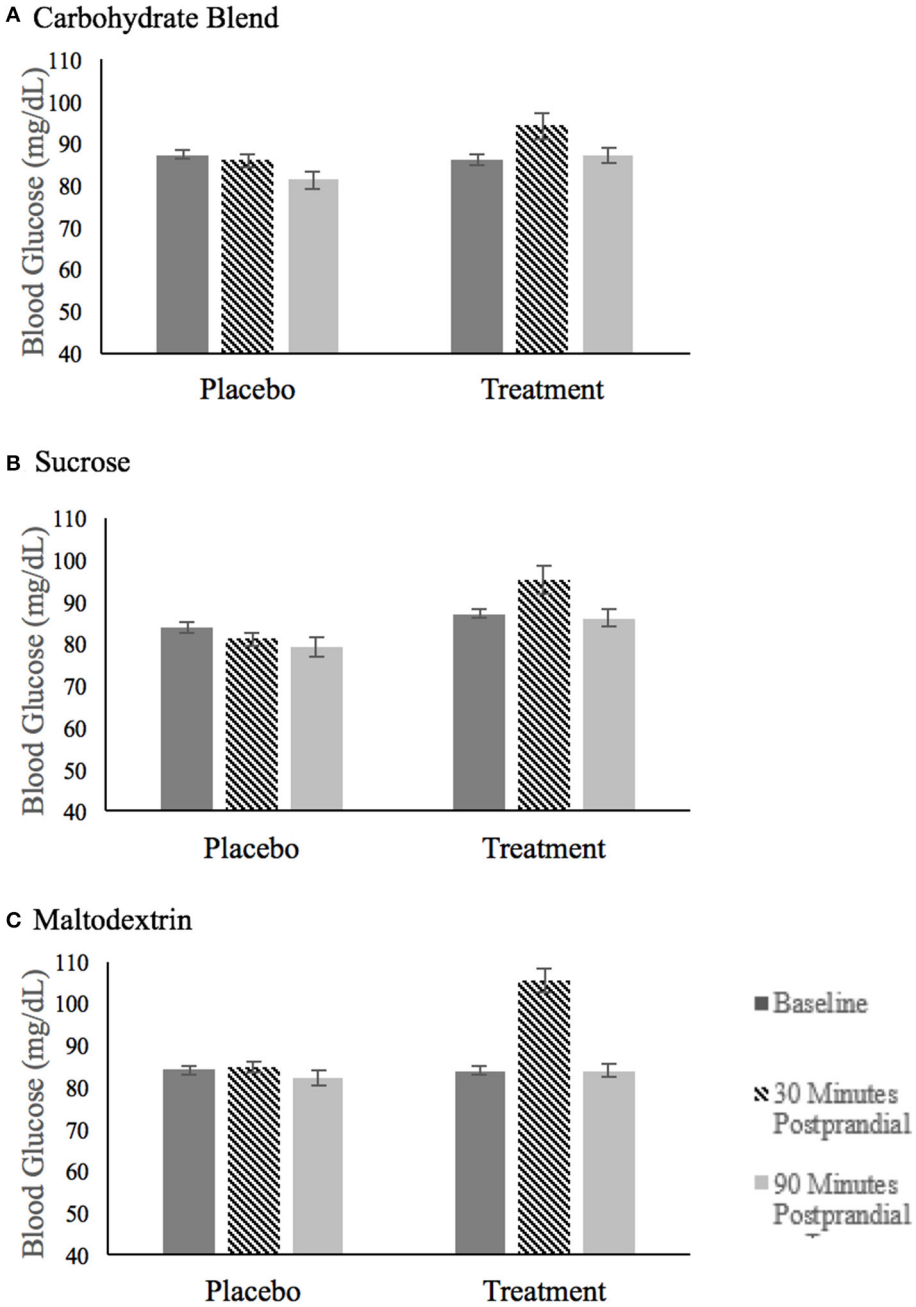
Fasting blood glucose after ingestion of the placebo and treatment drink at baseline (black bar), 30 min postprandial (hatched bar), and 90 min postprandial (light gray bar) for participants in the carbohydrate blend group **(A)**, the sucrose group **(B)** and the maltodextrin group **(C)**.

The blood glucose values for each treatment group are summarized in Figure 3. The analysis of blood glucose revealed a significant three-way group × condition × time interaction [*F*_(4, 144)_ = 4.666, *p* = 0.002, $\eta_{p}^{2}$ = 0.115]. This interaction was explored by examining blood glucose within each treatment group with baseline values included as covariates. This analysis revealed that there were no significant condition or time effects of blood glucose on the carbohydrate blend or sucrose groups. However, there was a time × condition interaction in the maltodextrin group [*F*_(1, 26)_ = 9.272, *p* = 0.005, $\eta_{p}^{2}$ = 0.263] with participants' blood glucose significantly higher at 10 min postprandial (*m* = 105.5) compared to 60 min postprandial (*m* = 83.8) in the treatment condition only. As expected, there was no change in glucose over time in the placebo condition (*p* = 0.387).

### Neurocognitive data

**Figure 4:**
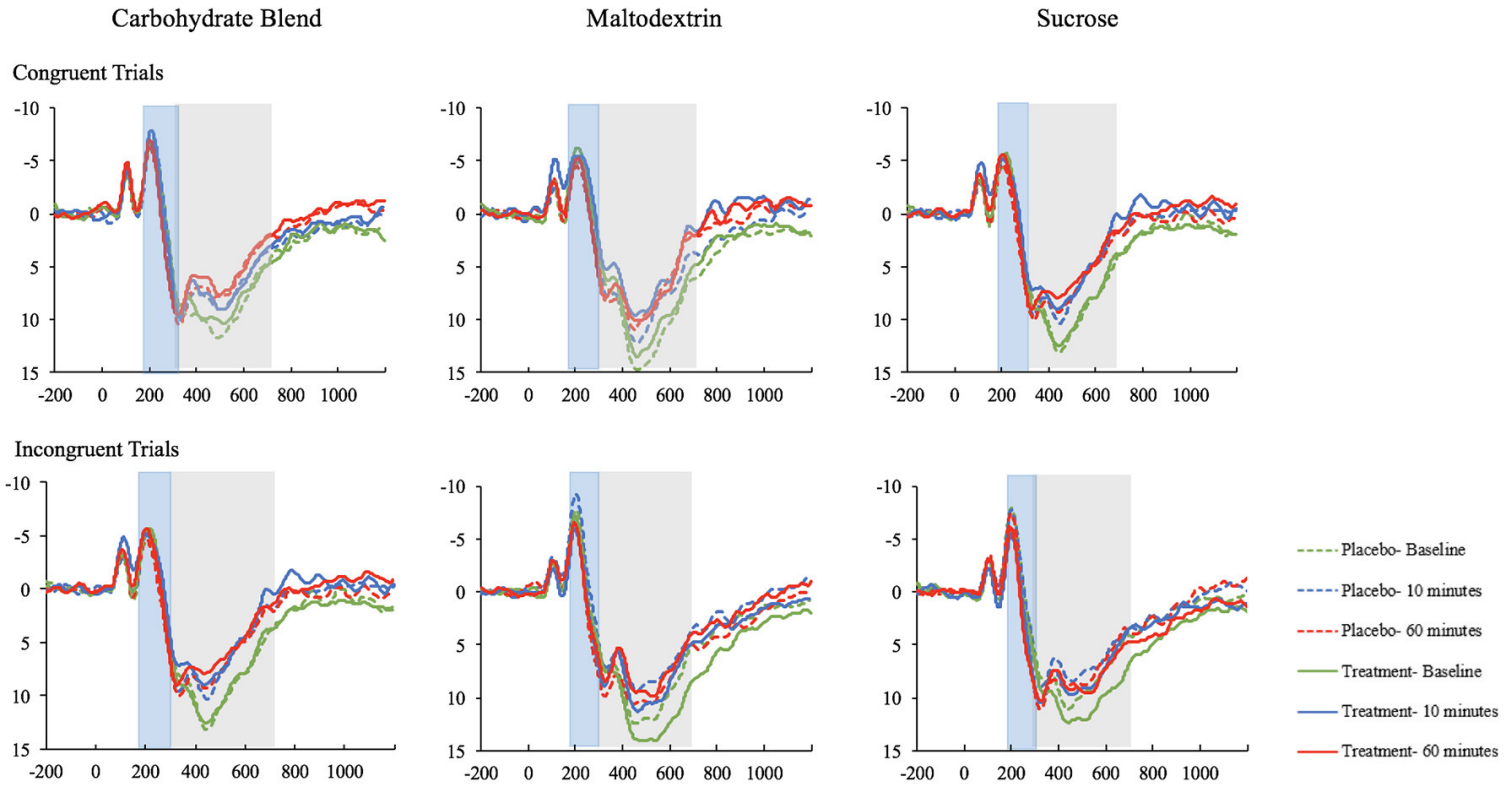
The ERP waveforms (ROI) for participants in the carbohydrate blend, matodextrin, and sucrose groups on the congruent and incongruent trials of the Flanker task. ERPs taken at baseline are depicted in green; ERPs at 10 min postprandial are depicted in blue; ERPs at 60 min postprandial are depicted in red; ERPs taken after ingestion of the placebo drink are depicted as dotted lines; ERPs taken after ingestion of the treatment drink are depicted as solid lines.

The effects most pertinent to our current research questions are those involving both time and condition. The covariates in both models were included to control for known variance that was expected to contribute to the overall model, and were therefore not examined individually. Thus, interaction effects involving time and condition, but excluding covariates will be the main focus of the following sections. The means with accompanying standard deviations can be seen in their entirety in the Table 3. The ERP waveforms for each treatment group at each time point can be seen in Figure 4.

#### Performance indices

##### Reaction time

For the Carbohydrate Blend group, no time × condition effects were found in the model adjusted or unadjusted for AUC. Likewise, in the Sucrose group, neither model showed any significant time × condition effects. For the Maltodextrin group, the reaction time analysis yielded no significant time × condition effects when left unadjusted for AUC. However, when AUC was adjusted into the model, an interaction between condition and time emerged [*F*_(1, 24)_ = 4.533, *p* = 0.044, $\eta_{p}^{2}$ = 0.159]. *Post-hoc* tests revealed that at 10 min postprandial, participants responded more slowly when undergoing the placebo condition (*m* = 586.9 ms) than when undergoing the treatment condition (*m* = 576.8 ms) (*p* = 0.027). There was no significant difference at 60 min postprandial as a result of condition (*p* = 0.738).

##### Performance accuracy

For the Carbohydrate Blend group, the model unadjusted for AUC revealed no pertinent significant effects for performance accuracy. However, when AUC was adjusted, a condition × time × congruency interaction was revealed [*F*_(1, 19)_ = 5.213, *p* = 0.034, $\eta_{p}^{2}$ = 0.215]. *Post-hoc* analyses revealed that in the placebo condition there was a main effect of congruency [*F*_(1, 19)_ = 4.929, *p* = *0*.039, $\eta_{p}^{2}$ = 0.206)] with higher accuracy for congruent stimuli (*m* = 93.1) compared to incongruent stimuli (*m* = 87.9). No significant main or interaction effects were found in the treatment condition. Furthermore, when examined by condition, neither the placebo or treatment condition showed any significant main or interaction effects involving the time factor. For the Sucrose drink, no time × condition effects were found in the adjusted or unadjusted models for accuracy. Likewise, for the Maltodextrin drink, no significant time × condition effects emerged for accuracy in the models adjusted and unadjusted for AUC.

#### N2

##### Peak latency

The analysis of the Carbohydrate Blend on N2 peak latency revealed a significant time × condition × congruency effect, but only when AUC was included into the model [*F*_(1, 19)_ = 13.314, *p* = 0.002, $\eta_{p}^{2}$ = 0.412]. Follow up analyses indicated no significant effects in the placebo condition, whereas in the treatment condition participants had later latencies for congruent (*m* = 267.2) compared to incongruent (*m* = 265.0) stimuli at 10 min postprandial (*p* = 0.013) and an opposite pattern with later latencies for incongruent (*m* = 259.0) compared to congruent (*m* = 254.6) stimuli at 60 min. No time × condition effects were seen for the Sucrose or the Maltodextrin groups when AUC was adjusted or unadjusted.

##### Peak amplitude

No effects regarding time × condition were seen in amplitude for any of the drink groups when AUC was left unadjusted. However, when AUC was adjusted in the model, a condition × time × congruency interaction emerged in the Maltodextrin group [*F*_(1, 24)_ = 8.104, *p* = 0.009, $\eta_{p}^{2}$ = 0.252]. This interaction was characterized by a time × condition interaction that existed for incongruent trials (*p* = 0.006) that did not exist for congruent trials (*p* = 0.787). At 10 min postprandial, there was a moderate effect of condition (*p* = 0.063) with larger (more negative) peaks in the placebo (*m* = −11.8) compared to the treatment (*m* = −9.9) condition whereas no difference was seen in at 60 min postprandial. No effects emerged for the Fiber Blend or Sucrose groups when AUC was adjusted.

#### P3

##### Peak latency

The analysis of P3 peak latency for the Carbohydrate Blend group revealed a significant interaction of condition × time × congruency [*F*_(1, 24)_ = 6.855, *p* = 0.015, $\eta_{p}^{2}$ = 0.222]. This effect was driven by a time × condition interaction that occurred for incongruent trials (*p* = 0.010) that was not present for congruent trials *(p* = 0.565). The time × condition interaction was the result of a difference between placebo and treatment conditions at 10 min postprandial, with participants having earlier latencies after ingesting the placebo drink (*m* = 436.6 ms) than after ingesting the treatment drink (*m* = 461.6 ms). The effect was not retained when AUC was included in the model.

In the Sucrose group, the analysis of peak latency revealed a significant condition × time interaction [*F*_(1, 23)_ = 7.438, *p* = 0.012, $\eta_{p}^{2}$ = 0.244]. This effect was driven by a marginal difference in latencies between conditions at 10 min postprandial, with participants' neural responses elicited earlier after ingesting the treatment drink (*m* = 440.3 ms) compared to the placebo drink (*m* = 452.7). There was no condition effect at 60 min postprandial.

In the Maltodextrin group, no condition × time effects were shown when AUC was adjusted or unadjusted.

##### Mean amplitude

**Figure 5:**
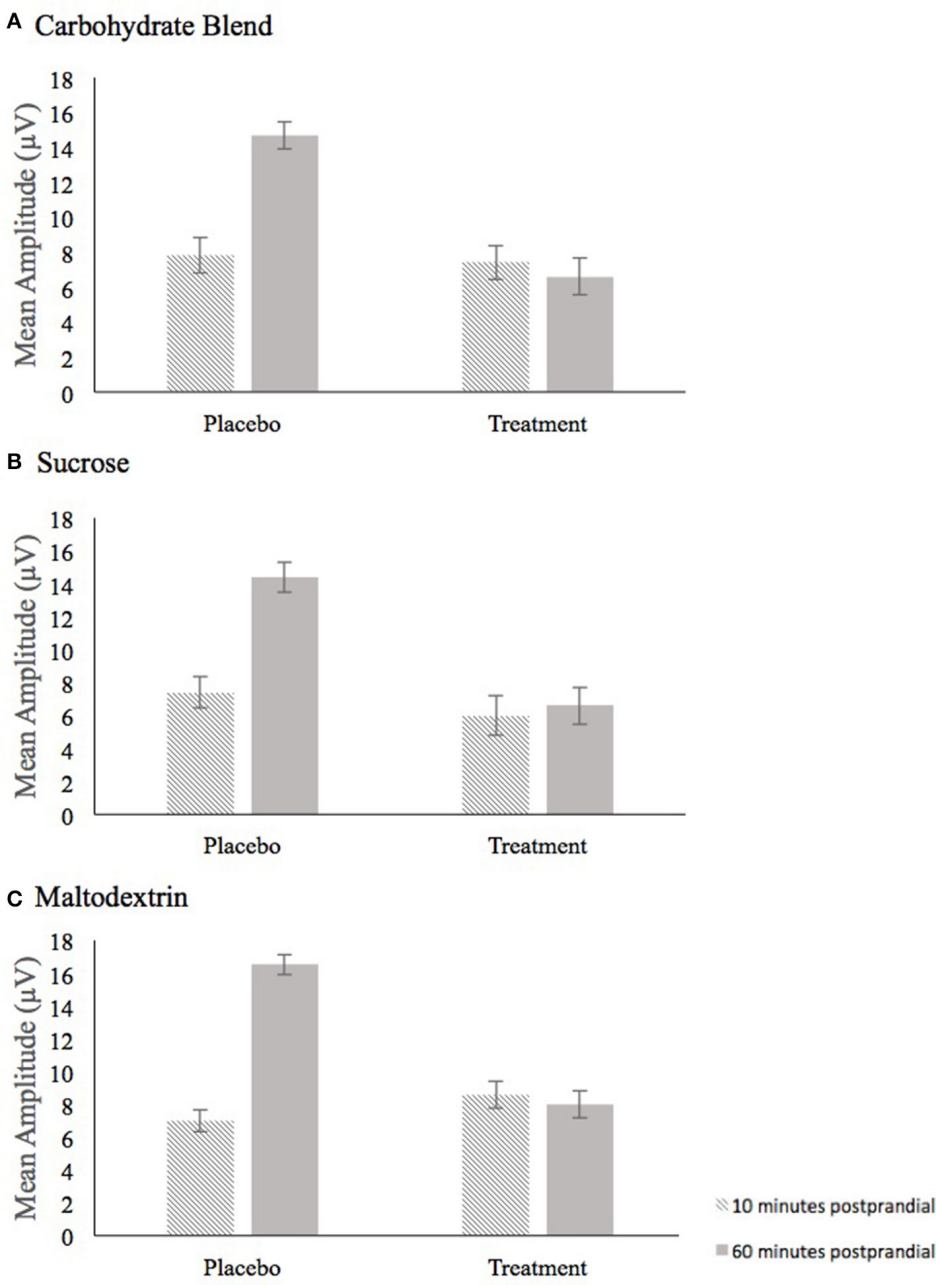
Depiction of the Condition × Time interaction for mean amplitude that persisted across all three drink conditions, carbohydrate blend **(A)**, sucrose **(B)**, and maltodextrin **(C)**. Amplitude at 10 min postprandial is depicted as the hatched bar; Amplitude at 60 min postprandial is depicted as the solid gray bar.

The analysis of P3 mean amplitude for the Carbohydrate Blend group revealed a condition × time interaction [*F*_(1, 24)_ = 8.995, *p* = 0.006, $\eta_{p}^{2}$ = 0.273] (Figure 5A). Follow up tests revealed that participants had higher amplitudes at 60 min postprandial (*m* = 14.7) compared to 10 min postprandial (*m* = 7.8) in the placebo condition only (*p* = 0.001), whereas there was no difference in the treatment condition (*p* = 0.778). This effect was not retained when AUC was included in the model.

In the Sucrose group, the analysis of mean amplitude revealed a condition × time interaction [*F*_(1, 23)_ = 6.368, *p* = 0.019, $\eta_{p}^{2}$ = 0.217] (Figure 5B) which indicated a significant difference in mean amplitude at 10 min postprandial (*m* = 7.4) and 60 min postprandial (*m* = 14.4) in the placebo condition only (*p* ≤ 0.001), whereas time was not a significant factor in the treatment condition (*p* = 0.345). This effect was not retained when AUC was included in the model.

For the Maltodextrin group, the analysis of mean amplitude revealed a condition × time interaction [*F*_(1, 30)_ = 13.010, *p* = 0.001, $\eta_{p}^{2}$ = 0.302] (Figure 5C) which was further characterized by a condition × time × congruency interaction [*F*_(1, 30)_ = 8.281, *p* = 0.007, $\eta_{p}^{2}$ = 0.216]. Follow up tests to the three-way interaction examined time and congruency in each condition and revealed a significant time effect in only the placebo condition (*p* = 0.002) with higher amplitudes at 60 min postprandial (*m* = 7.1) compared to 10 min postprandial (*m* = 16.5). No significant effects were shown among the time and congruency variables in the treatment condition (all *p*'s ≥ 0.070). This effect was not retained when AUC was included in the model.

## Discussion

The primary aim of the current study was to investigate the neurocognitive effects of three beverages with varying carbohydrate properties, which we investigated by examining the mean reaction time, performance accuracy, and N2 and P3 indices elicited during a modified flanker task. The secondary aim was to investigate the role of individual changes in blood glucose in the aforementioned effects, which we did by co-varying AUC in our statistical models. The study yielded several important findings, and the hypothesis that carbohydrate ingestion would facilitate behavioral and underlying ERP waveform measures elicited during an attentional inhibition task was met with the majority of measures employed in the study, although it was most robustly shown in the study of P3 amplitude. However, the hypothesis that the changes seen in our measures would be directly related to changes in blood glucose were only born out for the P3 measure. The specific details of our findings are summarized below.

Regarding reaction time, significant effects involving both the condition and time variables emerged only for the Maltodextrin group, and only when AUC was included in the statistical model. This suggests that while the numeric differences in reaction time between the treatment and placebo conditions were not sufficiently large to warrant statistical significance, suppressing the variance due to blood glucose change enhanced this effect. At 10 min postprandial, participants responded more quickly during the treatment condition compared to when undergoing the placebo. This finding supported our hypothesis that the performance indices would show improvement during the treatment condition, but did not support the hypothesis that the difference would be accounted for by changes in blood glucose. On the contrary, this difference only emerged when statistically controlling for blood glucose changes.

Regarding performance accuracy, significant effects involving condition and time were seen only for the Carbohydrate Blend group, and only in the model that accounted for AUC. However, *post-hoc* analyses indicated that this effect was driven largely by differences in response patterns for congruent and incongruent stimuli across conditions, an expected effect. Therefore, our hypothesis was not met regarding response accuracy; the results did not indicate that participants performed any better after ingesting the treatment drink compared with the placebo drink.

The results of the N2 analyses showed largely null effects. The only N2 amplitude effect was seen for the Maltodextrin group when AUC was adjusted. It was driven by a larger N2 at 10 min postprandial in the placebo condition compared to the treatment condition. This indicated an increase in neural resource allocation to complete the task in the absence of caloric intake, whereas the neural correlates of inhibitory control were stable when a carbohydrate was ingested. Therefore, these data did provide support of our hypothesis that performance would be facilitated after ingesting the treatment drink, although this was shown selectively for the maltodextrin group. It was also statistically shown only after adjusting for AUC, which did not support our hypothesis that changes in neural components would be directly related to blood glucose changes. Conversely, the effects appeared in the data only after accounting for blood glucose change, suggesting that corresponding changes in blood glucose served to mitigate this effect.

The only N2 effect for latency emerged in the Carbohydrate Blend group. It was a small difference numerically, that only emerged after controlling the variance due to blood glucose AUC, and appeared to be largely related to participants' reactions to congruent compared to incongruent stimuli after ingesting the treatment drink. Given that the blend consisted of Fibersol-2®–a resistant maltodextrin known to diminish glycemic response, suggests that blunting glucose availability using resistant carbohydrates may selectively facilitate neuroelectric indices of inhibitory control. However, to our knowledge this is the first study to directly examine the impact of carbohydrates on the N2. Therefore, additional research is necessary to confirm these findings. Further, the effects of the carbohydrate blend appear to be driven largely by differences in congruency, with participants responding earlier and with smaller amplitudes to congruent, rather than incongruent trials. This is a finding that has been robustly seen in prior literature using flanker tasks (Kopp et al., 1996; Heil et al., 2000), and suggests that in the incongruent trials of the flanker task there is a greater need for conflict monitoring, reflected in a larger peak and slower latencies for incongruent stimuli. Therefore, regarding N2, we did find evidence to support our hypothesis, although this evidence needs strengthened with further study.

P3 latency effects were present for the Carbohydrate Blend and the Sucrose groups. In both groups, these effects were due in large part to changes occurring in latencies at 10 min postprandial. In the case of the Carbohydrate Blend group, participants showed faster neural responses after ingestion of the placebo drink, whereas participants in the Sucrose group showed faster neural responses after ingestion of the treatment drink. Thus, our hypothesis that processing would be facilitated after ingestion of the treatment drink was confirmed for the Sucrose group, but not for the Carbohydrate Blend group. When AUC was adjusted in the statistical model, these effects were not retained, suggesting that any differences in neural processing speed were directly influenced by changes in blood glucose, confirming our secondary hypothesis that changes in P3 would be related to blood glucose.

The most robust effects, which also yielded the most straightforward interpretation, emerged in the analysis of P3 amplitude, for which all three treatment groups showed a remarkably similar pattern of elicitation. Across the groups, when AUC was not co-varied, participants had smaller P3 amplitudes that increased from 10 to 60 min postprandial when the placebo drink was ingested. However, when the treatment drink was ingested, P3 amplitudes remained largely stable. When AUC was added into the model, this effect was diminished. Thus, regardless of carbohydrate source, there was a significant increase in amplitude in the absence of caloric intake. In short, when participants were provided with a caloric load, their P3 amplitudes were sustained throughout the testing session; however, in the absence of calories, their P3 amplitudes exhibited a large increase over time, suggesting that participants were increasing neural resources to maintain performance. Furthermore, regardless of carbohydrate source, the increase in amplitude appears to be directly related to participants' blood glucose levels, suggesting that this effect was directly driven by the nutrient ingestion and not an uncontrolled source such as participant arousal. The P3 amplitude data provide strong evidence in support of both hypotheses.

Prior investigations into the neuroelectric consequences of acute caloric ingestion following an overnight fast have shown mixed results (de Bruin and Gilsenan, 2009) regarding P3 amplitude. Early studies showed that cognitive tasks completed after meal ingestion generally elicited larger P3 amplitudes (Geisler and Polich, 1990, 1992a,b). However, these studies were observational in that food intake on the day of cognitive testing was assessed via self-report questionnaires, groups were assigned *post-hoc*, the amount and type of food ingested was uncontrolled, and circadian effects were unaccounted. Several subsequent studies attempted to control for circadian effects by testing all participants in the morning and in a fasted state. Of these studies, one used a standard meal and found unaffected P3 amplitude after meal ingestion (Hoffman and Polich, 1998). Three studies used within-subjects designs in which the performance of the same participants was compared after they ingested drinks containing a glucose formulation and after a control drink. Of these, two found no change in P3 amplitude despite recording changes in blood glucose values (Geisler and Polich, 1994; Knott et al., 2001). Riby et al. (2008), on the other hand, showed smaller P3 amplitudes accompanied by shorter P3 latencies following ingestion of the glucose drink. Similarly, Hoffman et al. (1999) used a between-subjects design comparing P3 of a group given a dietary supplement after an overnight fast to a non-fasted group and found that the group given the dietary supplement showed marginally decreased P3 amplitudes although the effect failed to reach significance. Thus, despite early evidence suggesting that P3 may be modulated after nutrient ingestion, more controlled studies have suggested that, if anything, P3 may decrease in response to glucose.

The prior literature lends itself to two potential interpretations. Many have concluded, based on the abundance of null results, that the early work showing an increase of P3 amplitude following meal ingestion may likely be an effect of arousal or satiety, rather than on energy intake itself (Hoffman and Polich, 1998; Hoffman et al., 1999). Under this view, a larger P3 is more likely to be observed in participants who are not hungry and subject to circadian effects (Geisler and Polich, 1990, 1992b).

However, as Riby et al. (2008) suggest, the finding that P3 amplitude decreases after nutrient ingestion is not incompatible with what is known regarding P3. They conclude that glucose ingestion promotes more efficient neural processing, requiring the deployment of fewer cognitive resources in order to attain an equivalent behavioral effect. We believe the same process is at work in our data. When participants were deprived of glucose, peak amplitudes showed a compensatory pattern characterized by large increase over time in an effort to maintain performance over time. However, when participants were given a drink containing a carbohydrate source, their performance was stable, without an increase in the allocation of neural resources, as demonstrated by a stable P3 across the testing session. In fact, in the case of the sucrose group, performance appeared to be facilitated as they responded better to incongruent stimuli after the treatment drink, but this effect was selective for only participants receiving sucrose. Interestingly, when blood glucose AUC was accounted for, this effect did not retain statistical significance, suggesting that this effect is related to changes in blood glucose rather than to general arousal states or satiety. It should also be noted that none of the prior work investigating the neurocognitive effects of an acute nutritional intervention have studied this phenomenon in children. While this novel aspect of our study contributes to the existing literature, it is important to point out that P3 effects are known to change across development in terms of latency, amplitude, and topography (Johnstone et al., 1996; O'Connell et al., 2012). Furthermore, task demands may interact with these developmental trajectories for both N2 and P3 (Downes et al., 2017). Therefore, some of the ways in which our data differ from the ones that have been reported previously may be due to developmental differences. To parse out the developmental changes as they relate to acute nutritional paradigms, further research needs to be conducted using other types of cognitive tasks.

Our study was not without limitations. It is possible that overt behavioral performance effects (e.g., accuracy and reaction time) may have been elicited if we had administered a larger caloric load. All treatment beverages used in this current study provided approximately 150 kcal which represents approximately 50% of a typical meal consumed by children. Therefore, future work is needed to determine the extent to which caloric load impacts changes in neuroelectric function. Nevertheless, given that we observed differential effects on neuroelectric indices at a relatively low energy dose suggests that even modest intake of calories may support neurocognitive function following an overnight fast among preadolescent children. Another limitation of the current study was that the blood glucose sampling was limited to three time-points. Therefore, we were unable to comprehensively characterize the postprandial glycemic changes. Measurement of the complete trajectory of the glycemic response curve using a continuous glucose monitoring system, rather than the instantaneous glucose measurement, may have provided greater insight into our findings. These limitations notwithstanding, the strengths of the current study included a double-blind controlled design and group, measurement and statistical control of key covariates, and use of use of neuroelectric measures along with behavioral task outcomes.

In conclusion, our results point to a relatively general effect of intake of different sources of carbohydrate on acute changes in underlying neuroelectric function among preadolescent children. We contribute to the literature on this topic by demonstrating that the glucose facilitation effect extends to attentional inhibition and is supported by alterations in modulation of attentional resource allocation, as indicated by changes in the P3 amplitude. Further, we provide preliminary evidence for the potentially detrimental or compensatory neuroelectric mechanisms that characterize absence of energy intake or ingestion of artificial sweeteners such as sucralose. Future research is necessary to elucidate the extent to which caloric restriction or intake of artificial sweeteners impacts attentional control in preadolescent children.

## Ethics statement

This study was carried out in accordance with the recommendations of the Institutional Review Board of the University of Illinois with written informed consent from all subjects. All subjects gave written informed consent in accordance with the Declaration of Helsinki. The protocol was approved by the Institutional Review Board.

## Author contributions

AW conducted the ERP data reduction and analysis and wrote the first draft of the manuscript. LR conducted the behavioral data reduction and assisted in data collection and implementation of the study design. AK and NC provided contributions to the content and offered important feedback on the manuscript. NK and CH designed and supervised implementation of the study design as well as supervised and revised the manuscript.

### Conflict of interest statement

The authors declare that the research was conducted in the absence of any commercial or financial relationships that could be construed as a potential conflict of interest.

## Abbreviations

ERP: Event-related potential
EEG: electroencephalographic
EOG: electro-oculographic
ROI: region of interest.
